# Doctors in Fiction

**Published:** 1887-07-16

**Authors:** 


### Doctors in Fiction.

(*Continued from p. 252, vol. ii.*)

As unworthy of public confidence as Dr. Sangrado, though more fortunate in practice, is the peasant doctor of the old French *fabliau*, on which Molière founded his comedy of the "Médecin malgré lui." A princess is suffering from an abscess in the throat, and messengers are sent far and wide to look for doctors, who are promised magnificent rewards if they can cure her. One of the messengers halts at the cottage of the peasant Robert, and mentions his errand to Robert's wife, whose lord and master has the bad habit of beating her pretty frequently. As the royal messenger speaks of his mission a happy thought strikes the ill-used woman. "If my husband were beaten once or twice," she says to herself, "he would not be so ready to use his stick to me. Experience teaches mercy." Thereupon she tells the messenger that her husband is a physician of altogether exceptional skill, but so eccentric that he will not exercise his skill without the most severe compulsion ; every cure he effects must be beaten out of him. When Robert comes in, therefore, he is asked to go to court and cure the princess. He refuses, protesting that he is unable to do anything of the sort, whereupon such a shower of convincing blows falls upon him that he is willing to promise that he will go anywhere, do anything, if only he is let alone. He is literally beaten to court ; to every hesitation, every disclaimer of the power ascribed to him, the rod replies. Brought into the presence of the princess, he again declares his incompetence ; and again he is beaten till he cries for mercy. The contortions he makes while undergoing this infliction are so grimly quaint, that her highness, who is evidently not a princess of a tender heart, laughs so immoderately that the abscess in her throat bursts, and the desired cure is effected. But Robert's troubles are not over yet. Every sick person in the city wants to be cured by this marvellous physician, and he is beaten till he promises to attempt the relief of their diseases. He therefore gathers them all in a great hall, and solemnly assures them that it is essential for the cure of the others that the feeblest of them should be burnt to death, and his ashes swallowed by the rest. At this news the crowd, each individual of which had a moment before been vaunting the seriousness of his own case, suddenly discover that they are all perfectly well, and troop out in unseemly haste. Robert is thus left free to return to his home, but the story fails to inform us if his medical experience taught him to desist from the practice of wife-beating.

Perhaps not a few busy and worried doctors in these days would gladly adopt peasant Robert's cure for imaginary diseases, and yet these ailments have their value. Without them what would become of our health-resorts? It is not serious illness that fills the hotels and hydropathic establishments which arise all over the country in every spot where a ferruginous spring or air of special freshness gives excuse for the assembling of people in search of health. Those who go to such places are not really ill : they have lost strength and spirits in the fatigue of their daily work or amusement, and new scenes, new companions, new recreations, restore them to energy. The institutions exist for the convenient establishment of acquaintance and the carrying out of amusements. Cynics declare that they are much used as a British form of the Gallican matrimonial agency ; the doctor on the premises is not often called upon to attend to a serious case.

Such seems to have been the experience of Dr. Quackleben, the learned doctor who attended to the hypochondriacs, coquettes, schemers, and pleasure-seekers who frequented "St. Ronan's Well." Scott does not give us much proof of the doctor's skill : indeed, the doctor does not do so himself. He is, as his name suggests, more than half a charlatan ; and when he is not flattering Lady Penelope or evading Touchwood's cutting remarks, he is bent on winning the heart of Mrs. Blower, the well-endowed, albeit illiterate widow, who varies the doctor's name so often in speaking of him, that he indulges in no more than a fair requital when he changes hers once for all.

This is Scott's only large study of a medical man, though of late years medical women have been fain to point to Rebecca, the fair Jewess in "Ivanhoe," as a true portrait of their mediæval prototypes. In mediæval times the care of the sick seems to have been left greatly to women ; and there are not wanting authentic legends of fair nurses who then as now inflicted one wound while they healed another.

Thackeray gives us two portraits of doctors, the good and the bad. The first is kind Dr. Goodenough, who pulls scapegrace Pen through that sharp fever which brought him to the brink of the grave when he was staying with Warrington in Pump Court; and who showed himself so wise and thoughtful in getting foolish little Fanny away from Arthur's side when his mother's arrival brought the necessity for her self-imposed task of nursing to an end. An ideal physician this! Not merely skilful in his profession, but wise and tender in his ministry to restless brains and unhappy souls; knowing when to speak and when to be silent; not severe to human weaknesses, nor over-scornful of human prejudices; learned in that sympathetic knowledge of mankind which does not invariably accompany the most brilliant attainments in physiology and therapeutics. It is pleasant to know that the picture of Dr. Goodenough is drawn from a real doctor who tended the author as the doctor in the novel tended the hero; pleasanter still to think that there are but few of us who are not privileged to count a Dr. Goodenough among our own acquaintance.

*(To be continued.)*

